# Variola Vaccinæ or Vaccinia, or Cow-Pock

**Published:** 1838-08-15

**Authors:** N. Chapman

**Affiliations:** Professor of the Theory and Practice of Physic, in the University of Pennsylvania


### THE MEDICAL EXAMINER.

DEVOTED TO MEDICINE, SURGERY, AND THE COLLATERAL SCIENCES.

EDITED BY J. B. BIDDLE, M. D. AND M. CLYMER, M. D.

No. 17.] PHILADELPHIA, WEDNESDAY, AUGUST 15, 1838. [Vol. I.

### VARIOLA VACCINÆ OR VACCINIA, OR COW-POCK.

*By* N. Chapman, M. D., *Professor of the Theory and Practice of Physic, in the University of Pennsylvania.*

[Reported for this Journal.]

It is a very curious fact, showing how nicely balanced, in some instances, are the dispensations of Providence, that hydrophobia and vaccinia, the only diseases, perhaps, authentically derived from the brute creation, should exercise so different an influence over mankind. They are the antagonizing powers of evil and good—the one the cause of terror and death, and the other a source of comfort and the means of the preservation of life.

Confiding in accounts merely traditionary, or resting on authority quite as questionable, it might be concluded, that as a preventive of small-pox, the process of vaccination had been immemorially known in some of the oriental countries.

On its introduction into China from Europe, in 1810, such a claim to priority was at once preferred by one of their writers, who declares, that he finds the practice in a very ancient Chinese work. Cow-pox, he says, originated in a fly, which, fastening on the cow, sucks the blood, till it falls off; the blood of this fly, being then used to impart the disease, by inoculation, to the human species. More plausibly is it asserted, that the practice had been adopted for upwards of a century, in certain parts of England, Wales, and Ireland, antecedently to the formal promulgation of the fact. Entitled to still greater attention than these fabulous or vague reports, though also wanting in confirmation, is the averment that, in anticipation of this event, by a few years, a case of successful vaccination had been communicated to Sir George Baker, then a distinguished practitioner of London. Conceding, however, to it, and to all other pretensions, the credit which can reasonably be preferred, it is still manifest from such seminal hints, no material advantage was gained.

To Dr. Edward Jenner, of England, is due the honour of having introduced, and established, this momentous discovery, so interesting to medical science, and replete with benefits to mankind.

As might be supposed, he had in the commencement to encounter difficulties, and to resist various attempts, open and disguised, to despoil him of his well earned laurels. Convinced ultimately, however, of the validity of his titles, these were sanctioned by the British parliament, after the most deliberate investigation, and as a reward, he had voted to him the sum of thirty thousand pounds sterling.

In 1768, while Jenner was a student of medicine, residing in Gloucestershire, he heard of the prevalence of an affection on the udder of cows of a pustular nature, which occasionally infecting the hands of those who milked them, produced a correspondent affection, proving a preventive of small-pox, as well in the natural way, as by inoculation. It may not be uninteresting here to observe, that I have been told by Dr. Neal, of this city, that much about the same period, and certainly without any information from abroad, his father, who practised physic on the eastern shore of Maryland, was in possession of essentially similar facts in relation to this subject.

Deeply impressed with the value of the intelligence he had collected, Jenner repaired to London to prosecute his studies under the auspices of the celebrated John Hunter. During the period of their connection, frequent conversations were held between them, regarding the cow-pock, and its reputed properties. Engrossed, however, with other occupations, the preceptor was without leisure, or did not deem the matter worthy of his immediate attention. Having returned home, after a considerable interval, Jenner resumed his favourite investigation, which he steadily pursued, till he accomplished a discovery that has thrown around his name imperishable renown, and enrolled it with those—"Inventas aut qui vitam excolure per artis unique sui memores alios fecere merendo."

The points which he thought he had demonstrated were, that the cow-pock was readily imparted to the human species by inoculation, to be passed from individual to individual, without deterioration or change, giving an entire immunity against small-pox, it being itself a very mild affection, entailing no pernicious consequences, and utterly destitute of the quality of infection through the medium of the atmosphere, or of communication in any other mode than the one stated.

Entirely satisfied of the accuracy of his conclusions, he resolved to lay them before the public through the Transactions of the Royal Society of London. But that learned body placed no confidence in his representations, so extraordinary did they appear—and his paper was returned, with a friendly admonition to withhold it from the press, lest it should injure his reputation. Happily the advice was not followed—and in 1798, twenty years after the commencement of his inquiries, he published in a small pamphlet, in a style the most modest, his great discovery. From this period, the practice of vaccination came progressively to be diffused throughout the civilized world, meeting in different countries various degrees of resistance, according to the extent of prejudice against it, or the inefficiency of exertion in its behalf. Early adopted in the United States, and with little or no division of sentiment as to its utility, it was speedily spread over the wide expanse of our territories.

Of the origin and nature of the vaccine virus, we are not precisely informed. It has been seen, that Jenner first detected it in the udder of the cow.

But he advanced the conjecture, that a remoter source of it might be found in a sort of icherous humour of the horse's heel, vulgarly called the *grease*, which the cow received by milkers previously handling the feet of the horse. This explanation seemed to him the more probable, as it is customary in many parts of England for men to perform the double office of grooms and milkers. The account which he gives of this fluid is too curious to be omitted. He says, "the skin of the horse is subject to an eruption of a vesicular character, which vesicle contains a limpid fluid, showing itself most commonly in the heels. The legs, at first, become edematose, and then fissures are observable. The skin contiguous to these fissures, when accurately examined, is seen studded with small vesicles, surrounded by a small areola. These vesicles contain the specific fluid. It is the ill management of horses in the stable that occasions the malady to appear more frequently in the heel than other parts. I have detected it, connected with a sore in the neck of a horse, and on the thigh of a colt."

Experiments soon made by Coleman, the well known veterinary surgeon, led to an inference opposite to the one of Jenner, and which was supported by some very respectable authority. But Jenner in reply affirmed, that he had used the equine matter with complete success—and several others to whom he had transmitted it, as well as some deriving it themselves directly from the horse, declared that they had propagated from it the vaccine pustule in the human subject, capable of protection against small-pox. Granting these facts, his deduction from them is not warrantable. They only prove the identity of the virus in the horse and cow, and not at all the priority of the origin in either animal. Equally, almost, might a claim be set up for the goat, sheep, and poultry, in each of which the pustule is said to have been observed. Nor is there proof wanting of the susceptibility of the ass and the dog* to the disease, a pustule having been excited in each, supplying a virus causing the same affection in the human species.

As regards the disease in the cow, it was the opinion of Jenner, that it is merely a local affection, confined to the udder, showing itself chiefly on the nipples in the form of irregular pustules. These, at first, are commonly of a palish blue colour, or sometimes approaching to lividness, surrounded by a sort of erysipelatous inflammation, occasionally degenerating into phagedenic ulcers. The disease is of rare occurrence, and never appears except when cattle are collected in large herds, and then breaks out, at very uncertain periods among them, without any obvious or well ascertained cause. The infrequency and transient nature of its prevalence are such, that Jenner was constantly interrupted in his inquiries from the difficulty of procuring fresh supplies of matter. During the year 1828, the National Vaccine Institution of London, though they diligently sought it, could not hear of it in the cattle of England. As late, however, as 1830, it was reported to have been found in the cows of Italy, and again, two years afterwards, among those of India. Whether it is now to be met with in the United States is doubtful, though we have reason to believe it formerly existed in our cattle. There is no evidence within my knowledge, of its ever having been detected in our horses, or other domesticated animals.

* Jenner.

By Jenner and his immediate disciples, it was supposed, that the variolous and vaccine diseases were once the same, having received their present peculiarities by a transmission through the constitutions of the human and bovine races, for a long succession of time. The very title, *variola vaccinæ*, which he conferred, sufficiently expresses this hypothesis.

That horned cattle, at one period, were liable to an eruptive fever, bearing a close resemblance to variola, is rendered probable, at least, by some very curious facts, which seem to have been unknown to Jenner, or, at least, they are not mentioned by him. This disease, I believe, was first described by Fracastorius early in the sixteenth century,* as prevailing among cattle throughout the papal dominions, and to which he gave the title of lues bovilla. It is noticed by Ramazzini as recurring in 1690, and again in 1711, and also by Lancisi, each of whom has recorded it as existing in Lombardy. They endeavour to prove by quotations from the Latin poets, Lucretius, Virgil, Ovid, &c., that it was recognised even in such remote times. My limits forbid me to borrow more from these authorities, and which I the less regret, since their statements are fully confirmed by a more recent writer, who, perhaps, will claim greater credit.

My allusion is to a communication by Dr. Ledyard, to the Royal Society of London,† in 1780, from which it appears, that a similar disease, on different occasions, had broken out in England. But in 1745, and again in 1780, it very extensively pervaded that country. He says, "the disease in horned cattle, is an eruptive fever of the variolous kind: it bears all the characteristic symptoms, crisis, and event of the small-pox—and whether received by contagion, infection, or by inoculation, has the same appearances, stages, and determination, except more favourable by inoculation, and with this distinctive and decisive property, that a beast once having the sickness, naturally or artificially, never has it a second time." Contemporaneously, or nearly so, it prevailed in certain parts of France and Holland.

As recently as 1832, a disease very analogous is described as having broken out in a district of India, among cattle. "The animals appeared for a day or two dull and stupid. They were seized with a distressing cough, accumulation of phlegm in the mouth and fauces, and loss of appetite. On the fifth or sixth day, pustules made their appearance all over the body, especially on the abdomen, accompanied with fever and much general distress. These went on to ulceration, the hair falling off, wherever a pustule ran its course. The mouth and fauces, appeared to be the principal seat of the disease, being, in bad cases, one mass of ulceration, which impeded mastication, and proved fatal apparently from inanition. The mortality in this severe epizootic, was calculated at from fifteen to twenty per cent." ‡

* 1514.

† Transactions, 1780, part I.

‡ Cyclopædia of Prac. Med., vol. iv. p. 405.

It is to be regretted that no experiments were made, to determine more precisely the nature of the disease.

From these facts it has been conjectured with some plausibility, that as vaccinia had not existed, or been recognised long before Jenner's attention was attracted to the subject, it might have been the remains, in a mitigated state, of the former more general epizootic affection described by Ledyard. This inference is countenanced by the consideration, that epidemics become milder by lengthened continuance, and it is not inconceivable, that in this very case, what was originally a violent eruptive fever, may have finally been softened down into simple vaccinia.

Even stronger proof have we of the identity of the two diseases. Gesner informs us, that by inserting variolous matter into the udder of cows, a pustular affection was induced precisely like that of cow-pock, and we learn from Sunderland of Bremen, that on covering cows for several days with the sheets of patients under small-pox, the disease broke out in these animals. It has, too, been stated by Ozaman of Lyons, that if the variolous matter be mixed with fresh cow's milk, so as adequately to dilute its virulence, it induces, by inoculation, an eruption similar to that of the vaccine pustule, and, as conclusive of their identity, the virus taken from one of these pustules, causes the genuine vaccine affection, furnishing complete security against small-pox. To the accuracy of those reports, M. Robert, of Marseilles, has attested, and who adds, that a mixture of the variolous and vaccine matter with milk, will, at once, excite, on inoculation, the local vaccine pustule, without, as in the other instance, occasioning, at first, a more general eruption. Not having seen these publications, and indeed, only an incidental notice of them, and being wholly unacquainted with the authors, I am incapable of appreciating the evidence on which the statements rest, or the degree of personal authority to which the writers are entitled. They are, however, curious, and ought to be subjected to further and decisive experiments.

By those, on the contrary, who deny the primitive identity of the variolous and vaccine affections, it is urged, that independently of the striking difference in them, as they now exist, in general character and physiognomy, there are some facts, which give a downright refutation to the hypothesis.

1st. It is said, that variola is peculiar to the human species, all perfectly authenticated efforts to infect brute animals with it having failed. The late Mr. John Hunter, and some others more recently, it is understood, did not succeed in their repeated attempts to do it. Woodville, especially, an unquestionable authority, says, "that in the various attempts I have made to communicate the small-pox to animals, as dogs, rabbits, poultry, &c., both by the ordinary way of inoculation, and by injecting variolous matter into the veins, no disease was produced." * But we are assured by M. Vebourg, Professor in the Veterinary College at Copenhagen, that he communicated small-pox with virus taken from the human subject, to dogs, apes, and swine, and that the same had been effected at Berlin as to cows. These reports, it will be perceived, are inconsistent with those from similar experiments already cited, where the product was the vaccine, and not the variolous disease—and on a subsequent trial of covering cattle with the sheets of variolous patients fully impregnated with the contagious matter, it turned out an utter fallacy.

To decide, where statements are so contradictory, and we are without the opportunity of a cross-examination, to ascertain all those circumstances, so indispensable to the constitution of genuine testimony, is difficult. Circumstantial proof, where the chain is long, and each link confirmatory, is held in courts of criminal judicature to be entitled to greater weight than any single declaration, however positively made. But there is in philosophy a rule of evidence, more pertinent to this case, which alleges, that in all instances of prodigies, or wide deviations from the order of nature, or of events opposed to the tenor of general observation and experience, it is safer to conclude that the relater intentionally falsifies, or is deceived, than that such occurrences had actually taken place. Tried by this principle, we should be led to determine against the averments of the susceptibility of animals to the contagion of small-pox. Those domesticated or reclaimed, are nearly as much exposed to it as man himself, and who among us has ever known of any of them having been so infected? As in law, a man is presumed innocent till he is proved guilty, so in medicine, it were well, that no fact or doctrine should be received without the clearest demonstration of its verity, and by cumulative testimony.

2d. That the two diseases are not capable of intermixture, each preserving its peculiarities. Thus it is found, that if inoculation be performed with the two fluids blended, sometimes the vaccine, and at other times the variolous disease, will be excited, without the slightest change of character from the process.

3d. That when these fluids are inserted separately, though so contiguously that a common pustule is produced, either the one or the other disease will be raised by inoculation, according to the side of the pustule from which the virus may be taken.

4th. That the two fluids being introduced at the same time, each proves effective, to the evolution of the respective diseases. Yet to a certain extent, their actions are reciprocally restrained, the vaccine pustule is smaller and proceeds slower to maturity, while the variolous eruption suffers pretty much in the same way.

These are the arguments by which the opposite views on this point are vindicated. The question may be considered still as *sub judice*, left open for decision on further and better evidence!

In the practice of vaccination, certain rules should be carefully observed, the first of which regards the period of life. Except under very urgent circumstances, the operation is not to be performed sooner than a month after birth. "The uncertainty of organization being complete, and the extreme delicacy and irritability of the new-born babe, are the grounds usually assigned for this advice." It can scarcely be doubted, that at so early an age, the disease is less readily imparted, and, perhaps, from the great constitutional changes which belong to

* Woodville's History of Inoculation, note, p. 3.

this period of existence, the vaccine impression when received, may be more liable to be weakened or entirely subverted.

